# Group B *Streptococcus* infection-induced ovarian vein thrombosis identified during cesarean section: A case report and a literature review

**DOI:** 10.1097/MD.0000000000034141

**Published:** 2023-06-23

**Authors:** Jianqiong Li, Meifang Zhou, Chaoman He, Fengbing Liang

**Affiliations:** a Department of Obstetrics and Gynecology, Sir Run Run Shaw Hospital, School of Medicine, Zhejiang University, Hangzhou, China; b Key Laboratory of Reproductive Dysfunction Management of Zhejiang Province, School of Medicine, Zhejiang University, Hangzhou, China.

**Keywords:** Group B *Streptococcus*, ovarian vein thrombosis, placental abruption, pregnancy, stillbirth

## Abstract

Ovarian vein thrombosis (OVT) is a rare yet potentially life-threatening condition associated with thromboembolic events. Group B *Streptococcus* (GBS) is a type of β-hemolytic Gram-positive bacterium known for asymptomatic colonization in the lower genital and gastrointestinal tracts. Here we reported a 35-year-old multiparous woman with gestational diabetes who suffered from placental abruption, stillbirth, OVT, septic shock, and renal failure due to severe GBS infection.

**Patient concerns:**

A 35-year-old woman with gestational diabetes presented with acute and sustained lower abdominal cramping, vaginal bleeding, and fever at 35 gestational weeks.

**Diagnoses:**

Based on preoperative ultrasound and intraoperative findings, the patient was diagnosed with placental abruption, intrauterine fetal demise, and right OVT. GBS was cultured from the amniotic fluid obtained during cesarean section.

**Interventions:**

The patient underwent a right adnexectomy during a cesarean section and received intravenous antibiotics. Subsequently, an ultrasound-guided uterine curettage was performed due to recurrent fever.

**Outcomes:**

After a prolonged course of intravenous antibiotics for over a month, the patient recovered and was discharged from the hospital.

**Lessons:**

This case underscores the need for early initiation of anticoagulant protocols in cases of OVT, particularly when GBS infection is identified as a predisposing factor. Further research and awareness are warranted to better understand the relationship between GBS infection and OVT and to optimize management strategies in such cases.

## 1. Introduction

Ovarian vein thrombosis (OVT), an infrequent yet potentially severe condition, often occurs in association with pregnancy or delivery, pelvic inflammation, abdominal malignancies, and pelvic surgery. Notably, while deep venous thrombosis is a concerning condition with an incidence rate of 1.6%, the rarity of OVT at 60-fold less frequency highlights the critical importance of recognizing and promptly diagnosing this condition in at-risk patients.^[1]^ Most cases of OVT present with postpartum fever and abdominal pain, with the right side being the predominantly affected (70%–80% on the right and 10% bilateral involvement) due to the rightward rotation of the uterus and anatomical factors.^[2]^ Complications of OVT include secondary pulmonary embolism, which occurs in approximately 2% of cases and typically involves small emboli.^[3]^ In rare cases, thrombosis extends to the renal vein or involves freely floating thrombi in the inferior vena cava.^[4–7]^ Recurrence rates are 6.1% at 1 year and 14.3% at 5 years, with active cancer being a common factor.^[1]^ Standard treatment for OVT typically involves the use of low molecular weight heparin or warfarin for 1 to 3 months.^[2,8]^

Pregnancy, a physiological state characterized by profound changes in hemostasis, confers an increased susceptibility to venous thromboembolism due to the multifaceted effects of Virchow triad, encompassing hypercoagulability, venous stasis, and endothelial injury.^[9]^ Endothelial injury, a critical component of this triad, can occur during obstetric procedures such as vaginal and cesarean deliveries, and from uterine infections, further exacerbating the prothrombotic milieu associated with pregnancy.^[10]^

Group B *Streptococcus* (GBS), also known as *Streptococcus agalactiae*, is a β-hemolytic Gram-positive bacterium that colonizes the lower genital and gastrointestinal tracts of individuals asymptomatically. The prevalence of GBS colonization varies geographically, with rates ranging from 1 in 3 pregnancies in the Caribbean to 1 in 6 in South and East Asia and a global average of approximately 18%.^[11]^ When GBS travels up from the vagina to the uterus, urinary tract, or bloodstream, it can cause severe clinical outcomes, such as miscarriage, preterm labor, stillbirth, chorioamnionitis, pyelonephritis, and sepsis. Additionally, neonates born to GBS-colonized mothers are at risk of developing invasive infections, such as pneumonia, sepsis, and meningitis, with an incidence of about 1%.^[12]^

This report presented a case study of a 35-year-old multiparous woman with gestational diabetes who suffered from placental abruption, stillbirth, OVT, septic shock, and renal failure due to severe GBS infection. The patient provided written consent for the publication of her case, and the institutional review board of our hospital confirmed that this case report was exempt from the requirement for approval.

## 2. Case presentation

### 2.1. Patient information

At 35 weeks and 4 days gestation, a 35-year-old Chinese woman presented to a local maternity hospital with lower abdominal pain, vaginal bleeding, and fever. The patient had a history of 2 live births, both delivered by cesarean section, and had been previously diagnosed with gestational diabetes during her second pregnancy. She reported good compliance with her diabetes management and had been monitoring her blood glucose levels regularly. Upon further questioning, the patient revealed that she was self-employed. She reported no known exposure to hazardous substances or environmental toxins. The patient also reported that she had remarried following her second cesarean section and was currently experiencing some marital stress. She denied any use of tobacco, alcohol, or illicit drugs.

### 2.2. Initial treatment

#### 2.2.1. Admission to a local maternity hospital.

**Figure 1.**
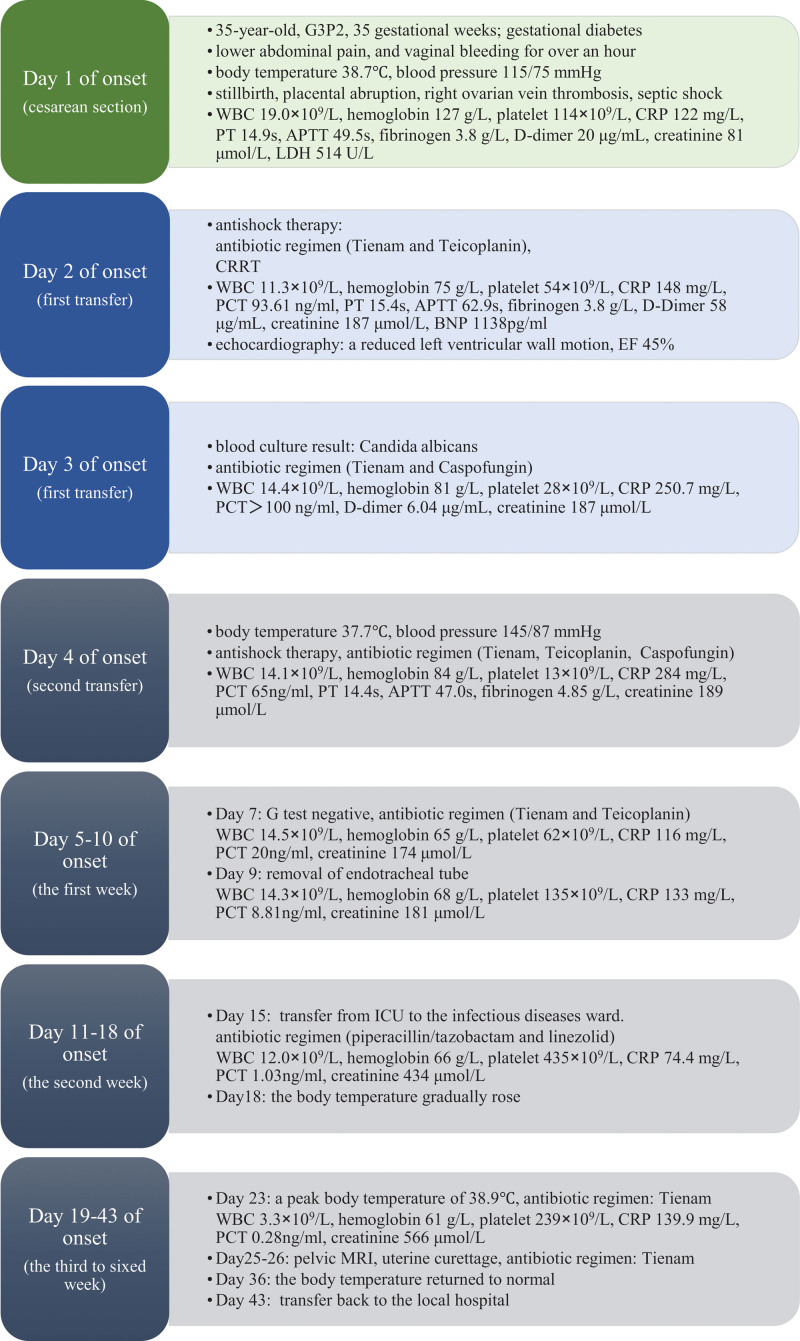
Timeline of events and findings. APTT = activated partial thromboplastin time, BNP = brain natriuretic peptide, CRP = C-reactive protein, CRRT = continuous renal replacement therapy, EF = ejection fraction, ICU = intensive care unit, LDH = lactate dehydrogenase, MRI = magnetic resonance imaging, PCT = procalcitonin, PT = prothrombin time, WBC = white blood cell count.

During the patient initial examination, her vital signs were obtained, revealing a pyretic state of 38.7°C, a pulse of 124 beats per minute, and normal blood pressure. She presented with sustained, strong contractions and a board-like abdomen, prompting further investigation *via* a fetal ultrasound. The ultrasound showed a biparietal diameter of 8.8 cm, indicating stillbirth. A lower limb venous ultrasound was performed, yielding negative results for thrombosis. Corresponding laboratory results are detailed in Figure 1. After suspecting placental abruption, a cesarean section was performed as a precautionary measure. During the surgical procedure, a definitive diagnosis of placental abruption was established based on the visualization of a substantial retroplacental hematoma.

Furthermore, the right adnexa exhibited a congested, dark, and swollen appearance with distended ovarian veins, suggesting right OVT. The patient underwent a right adnexectomy to address the suspected thrombotic event. During the cesarean section, the patient clinical presentation raised concern for septic shock, as evidenced by a fever of high magnitude and tachycardia reaching 180 beats per minute, prompting the administration of intravenous Tienam (1.0 g). After the surgical intervention, the patient exhibited coagulation dysfunction necessitating the administration of fresh frozen plasma (820 mL), and was subsequently transferred to an alternative hospital for ongoing treatment.

#### 2.2.2. Transfer to the intensive care unit (ICU).

Upon admission to the new hospital, the patient was promptly transferred to the ICU, where she received mechanical ventilation, sedation, and analgesia. A contrast-enhanced computed tomography scan was conducted, revealing bilateral pulmonary infiltrates and increased uterine size. Laboratory results showed elevated levels of C-reactive protein, procalcitonin, D-dimer, and brain natriuretic peptide. Coagulation abnormalities manifested as prolonged prothrombin time and activated partial thromboplastin time, indicating possible disseminated intravascular coagulation. Additionally, the patient exhibited reduced left ventricular wall motion and an ejection fraction of 45%, suggestive of cardiac dysfunction. The patient is currently receiving a combination of broad-spectrum antibiotics, norepinephrine to maintain hemodynamic stability, transfusions of packed red blood cells, fresh frozen plasma, and albumin to manage hypoalbuminemia. Continuous renal replacement therapy has also been initiated to manage anuria.

#### 2.2.3. Transfer to a specialized facility.

The patient condition deteriorated on the fourth day of hospitalization, prompting referral to our specialized facility. Invasive catheters were placed to enable close monitoring and management of her condition. A CT scan revealed increased uterine density and patchy high-density shadows in the right adnexal area, suggestive of potential hemorrhage. Further analysis of the amniotic fluid obtained during the cesarean section showed the presence of GBS. Notably, comprehensive laboratory investigations, including antiphospholipid antibody, lupus anticoagulant, antinuclear antibody, antithrombin III, protein C, and protein S assays, all yielded negative results. A collaborative multidisciplinary team convened to deliberate on the optimal management approach, resulting in the adjustment of the anti-shock and anti-infection regimens in light of the patient evolving clinical status. Finally, on the ninth day post-onset, the endotracheal tube was removed.

### 2.3. Uterine curettage

**Figure 2.**
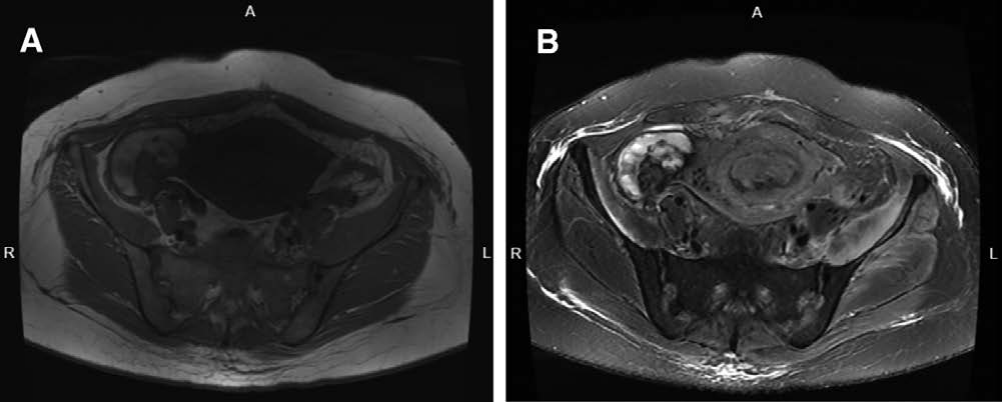
T1- and T2-weighted MRI images of a tortuous strip on the right side of the pelvis. (A) A short T1 signal shadow is observed on the tortuous strip. (B) The same tortuous strip displays a short T2 signal shadow. MRI = magnetic resonance imaging.

**Figure 3.**
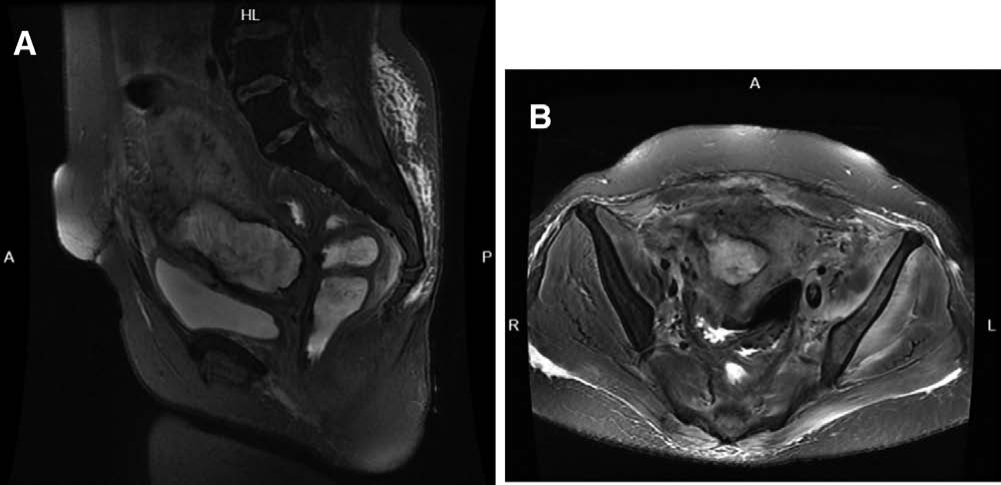
A small notch-shaped extension from the thinner anterior wall of the right lower segment of the uterine cavity to the serosa exterior. (A) A transverse section image. (B) A sagittal section image.

The patient was transferred from the ICU to the infectious disease ward on the fifteenth day post-onset. Despite initial improvement, she experienced a recurrence of fever on the eighteenth day after onset, which peaked at 38.9°C. Pelvic magnetic resonance imaging revealed massive intracavitary bleeding, a defect in the lower anterior wall of the uterus (Fig. 2), and a hematoma or thrombosis of the right ovarian vein (Fig. 3). Subsequently, ultrasound-guided uterine curettage was performed, with the removal of blood clots and a 4 × 3 cm tissue from the uterine cavity. A 3-way urinary catheter was then placed in the uterine cavity for drainage, and subsequent drainage volumes measured 80 mL, 20 mL, and 30 mL, respectively. The catheter was removed on the fourth day post-operation. Routine pathology following the procedure revealed a large amount of necrotic tissue. The patient received tigecycline for 10 days post-curettage, and her body temperature eventually normalized.

### 2.4. Follow-up care

One week after the patient body temperature normalized, she was transferred to her local hospital, where she received meticulous care and 18 days of treatment. During this period, she underwent a course of intravenous anti-infective therapy with piperacillin-tazobactam and did not experience any recurrence of fever, abdominal pain, or abnormal vaginal bleeding. Her hemodialysis regimen was gradually adjusted from thrice weekly to once weekly, and her creatinine levels consistently declined over time.

About 5 months after the onset of her illness, the patient underwent a comprehensive reevaluation. Gynecological ultrasound findings revealed no abnormalities, and her inflammatory markers remained within the normal range. Her daily urine output was approximately 1000 mL, and her creatinine levels remarkably declined to 300 μmol/L. Presently, the patient reports only a reduced appetite, which is approximately half of what it was prior to her illness, and a mild weight loss, resulting in a body mass index of around 18.4 kg/m^2^. Despite these symptoms, the patient can independently managing her daily activities with ease.

## 3. Discussion

This case report presented a 35-year-old multiparous woman who developed placental abruption, stillbirth, OVT, and renal failure due to severe GBS infection. The patient clinical presentation was notable for several established risk factors for maternal sepsis,^[13]^ including advanced maternal age (>35 years old), GBS colonization, impaired glucose tolerance, and a history of cesarean delivery. Despite the rarity of severe maternal and neonatal outcomes caused by GBS infection in coastal cities in China, our case highlights the importance of recognizing and promptly treating this potentially life-threatening condition.

GBS exhibits a dual nature as both a commensal microbe and a highly virulent pathogen.^[14]^ Although colonization by GBS is generally benign, the organism can cause severe damage to the host if it invades niches beyond the gastrointestinal and vaginal mucosae leading to severe clinical outcomes in pregnant women and neonates. Both bacterial factors and host-mediated mechanisms influence the transition of GBS from commensal to pathogenic origin.

Despite the global burden of stillbirths, there has been a lack of attention and reporting on the impact of GBS infections. It was not until 2017 that the global evaluation of GBS-related stillbirths was published,^[15]^ revealing a 4% incidence in Africa and a 1% incidence in developed regions. While there have been 65 reported cases of GBS-related stillbirths since 2020, there is a lack of data on this issue in Asia.^[15]^ Notably, GBS-related stillbirths occur 50% of the time between 20 and 28 weeks of gestation, and the remaining 50% occur after 28 weeks.^[16]^ Given these alarming statistics, it is imperative to fully recognize the severe outcome of GBS infections, even in cases of asymptomatic colonization. As such, effective GBS vaccines should be considered a potential intervention to prevent stillbirths, particularly in low- and middle-income settings.^[17,18]^

During our patient treatment, uncertainties regarding optimal management strategies have arisen, which we aim to address herein.

*Is it necessary to perform surgical excision of the affected ovary?*

In cases of postpartum OVT, the standard treatment typically involves anticoagulation and antibiotics without mention of surgical intervention. Existing surgical literature often details scenarios where anticoagulation is contraindicated or when conventional antimicrobial or anticoagulation therapies have been unproductive. However, there is limited documentation on the discovery of OVT during surgery, raising concerns about the necessity of surgical management of OVT with discoloration and swelling. Additionally, uncertainties remain regarding whether the ovary discoloration and swelling indicate necrosis and the potential complications of retaining the ovary with thrombosis. To address these knowledge gaps, we reviewed relevant literature on OVT refer and consulted the literature on ovarian torsion to provide insights into these questions. Mandelbaum *et al*^[19]^ undertook a comparative investigation of perioperative complications in women under 50 years old with ovarian torsion who received either conservative surgery or oophorectomy. The proportion of conservative surgeries increased from 18.9% to 25.1% between 2001 and 2015 in the United States. The findings of the study showed that conservative surgery was associated with a 30% lower risk of overall perioperative complications and did not increase the risk of venous thromboembolism or sepsis. Geimanaite et al^[20]^ conducted a long-term follow-up study on 20 girls and found that conservative surgical management of ovarian torsion in children was safe and preserved normal ovarian anatomy and function after detorsion and retention of the ovary in the abdominal cavity. Additionally, literature reviews^[21–23]^ support ovarian detorsion as the preferred option for managing ovarian torsion over oophorectomy. Based on the present findings, it is postulated that anticoagulant therapy may be a viable option to restore ovarian blood supply and reduce complications in cases where OVT is detected during surgery. The objective of the present study was to determine the causal factors behind the decision for a right adnexectomy and the subsequent development of septic shock during cesarean section. It remains unclear whether the septic shock arose from infectious thrombosis of the right ovarian vein, thereby prompting the decision for adnexectomy, or if other factors were involved. If adnexectomy is not feasible, local control of the infection in the pelvic cavity may be a viable alternative. More evidence is needed to guide the surgical indications for OVT.

*What was the rationale behind the omission of anticoagulation therapy?*

As per established guidelines, anticoagulation therapy and anti-infective therapy should be implemented concurrently in patients with recurrent fever and OVT. In our case, the decision to forgo anticoagulation therapy was influenced by several factors, including the patient unstable vital signs, low hemoglobin, and platelet levels when transferred to our hospital (Fig. 1), the potential risk associated with contrast-enhanced CT or magnetic resonance imaging scans due to renal insufficiency. Additionally, we lacked comprehensive information on the initial surgical procedure performed at another hospital, and the possibility of bleeding-related hematoma in the right adnexal area could not be excluded. While such factors contributed to our reluctance to perform anticoagulation therapy, it is imperative to acknowledge that infection-induced endothelial damage promotes thrombosis, which can exacerbate the complexity of infections. In light of our findings, we recognized that earlier initiation of anticoagulation therapy might be beneficial in preventing further complications.

## 4. Conclusion

The case study herein highlights the significance of exploring the correlation between GBS infection and OVT and underscores the necessity for more epidemiological data, including maternal and fetal outcomes of GBS in Asian populations, as well as optimal management strategies of OVT.

## Author contributions

**Conceptualization:** Jianqiong Li, Meifang Zhou.

**Data curation:** Chaoman He.

**Writing – original draft:** Jianqiong Li.

**Writing – review & editing:** Fengbing Liang.
